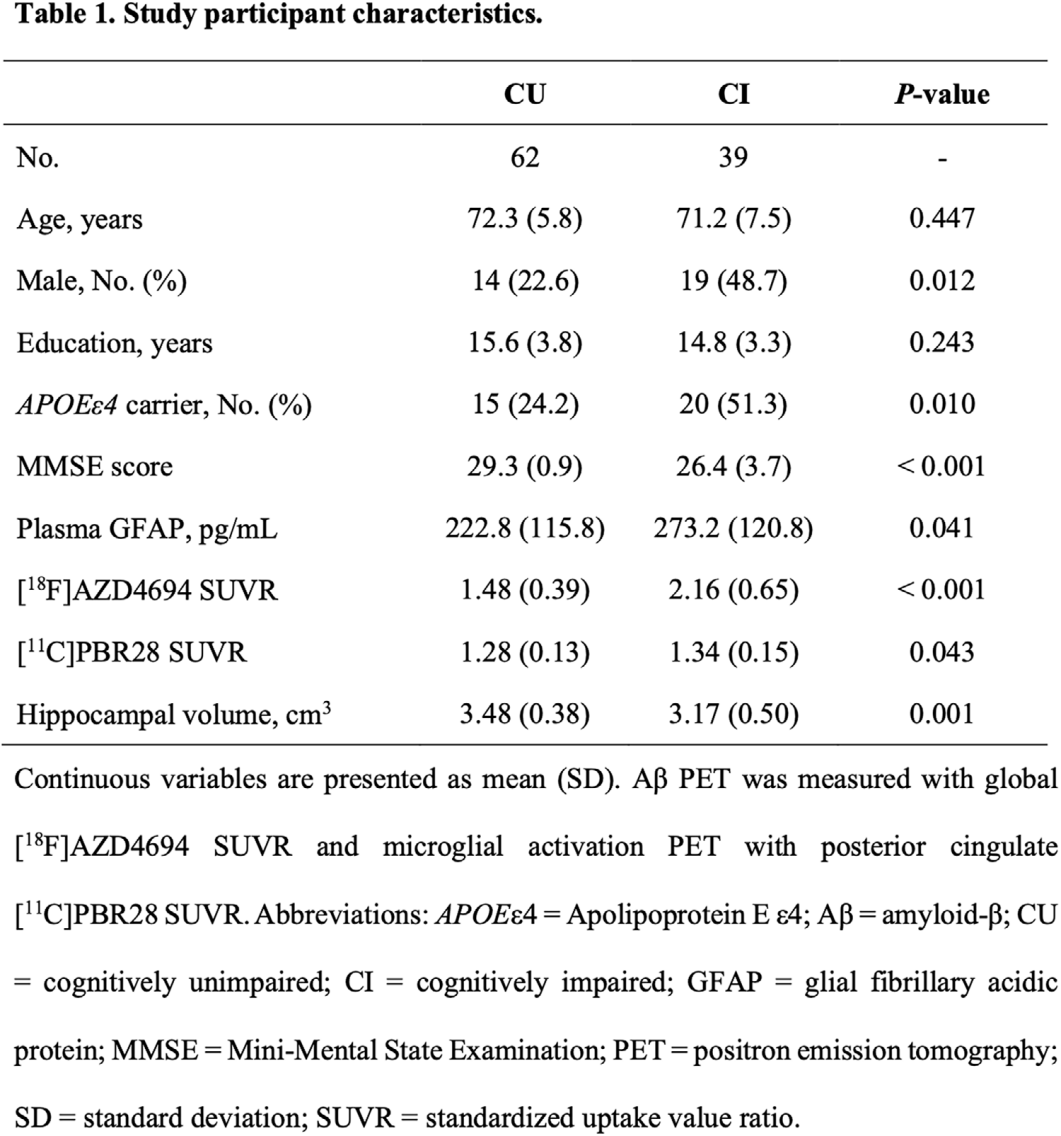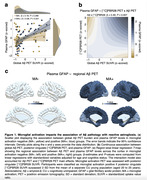# Microglial activation impacts amyloid‐β effects on reactive astrogliosis

**DOI:** 10.1002/alz.094387

**Published:** 2025-01-09

**Authors:** João Pedro Ferrari‐Souza, Guilherme Povala, Bruna Bellaver, Pamela C.L. Ferreira, Firoza Z Lussier, Douglas Teixeira Leffa, Cristiano Schaffer Aguzzoli, Carolina Soares, Giovanna Carello‐Collar, Wyllians Vendramini Borelli, Joseph Therriault, Nesrine Rahmouni, Arthur C. Macedo, Stijn Servaes, Jenna Stevenson, Serge Gauthier, Diogo O. Souza, Lucas Porcello Schilling, Mychael V Lourenco, Dana Tudorascu, Pedro Rosa‐Neto, Tharick A. Pascoal, Eduardo R. Zimmer

**Affiliations:** ^1^ Universidade Federal do Rio Grande do Sul, Porto Alegre, Rio Grande do Sul Brazil; ^2^ University of Pittsburgh, Pittsburgh, PA USA; ^3^ Brain Institute of Rio Grande do Sul, PUCRS, Porto Alegre, RS Brazil; ^4^ Universidade Federal do Rio Grande do Sul, Porto Alegre, RS Brazil; ^5^ McGill University, Montreal, QC Canada; ^6^ Universidade Federal do Rio Grande do Sul, Porto Alegre Brazil; ^7^ Universidade Federal do Rio de Janeiro, Rio de Janeiro Brazil

## Abstract

**Background:**

Glial reactivity is a key phenomenon in Alzheimer’s disease (AD) and is closely associated with amyloid‐ß (Aß) pathology. Although compelling experimental data suggest that microglial activation modulates reactive astrogliosis, it remains to be elucidated whether microglial activation influences the association of Aß pathology with reactive astrogliosis in the living AD human brain. Here, we tested the association of microglial activation and Aß pathology with reactive astrogliosis in individuals across the aging and AD clinical spectrum.

**Method:**

We studied 101 participants (62 cognitively unimpaired [CU], 26 with mild cognitive impairment [MCI], and 13 with AD dementia) from the Translational Biomarkers in Aging and Dementia (TRIAD) cohort. Individuals had available positron emission tomography (PET) for Aß ([18F]AZD4694) and microglial activation ([11C]PBR28), as well as magnetic resonance imaging. We further assessed reactive astrogliosis with plasma glial fibrillary acidic protein (GFAP). Linear regression analyses were used to investigate the associations between Aß, microglial activation and GFAP.

**Result:**

Demographic characteristics of the study population are reported in Table 1. Regression analyses revealed a significant positive association between Aß PET burden and plasma GFAP levels in microglial activation‐positive but not in microglial activation‐negative individuals (Fig. 1A). A significant interaction between continuous values of Aß PET burden and [11C]PBR28 PET uptake on plasma GFAP levels (Fig. 1B) supported that microglial activation affects the association of Aß pathology with reactive astrogliosis. Analysis of variance further confirmed that the model with the interaction term was the most adequate to describe the association of Aß PET and microglial activation PET with plasma GFAP (P = 0.007). In additional analyses investigating the topography of the observed findings, we found that higher Aß PET burden was associated with higher plasma GFAP levels only in the presence of microglial activation positivity across Aß‐vulnerable cortical brain regions (Fig. 1C).

**Conclusion:**

Our results suggest that microglial activation impacts Aß‐dependent reactive astrogliosis in the living AD brain. This can help to better understand the complementary roles of glial cells in neurodegenerative diseases, as well as provide insights for the development of novel therapeutic strategies for AD targeting the interplay between Aß and glial reactivity.